# Real‐World Performance of CSF Kappa Free Light Chains in the 2024 McDonald Criteria

**DOI:** 10.1002/acn3.70300

**Published:** 2026-01-07

**Authors:** Maya M. Leibowitz, Ryan Cooper, Stefania Kaninia, Kathryn I. Challis, Will Greenway, Maria Bonello, Mahmoud Elbahnasawi, Deborah T. Shode, Dominika Gajdasik, Victor Iliev, Alison M. E. Whitelegg, Ian Galea

**Affiliations:** ^1^ Clinical Neurosciences, Clinical & Experimental Sciences, Faculty of Medicine University of Southampton Southampton UK; ^2^ Wessex Neurological Centre University Hospital Southampton NHS Foundation Trust Southampton UK; ^3^ Clinical Biochemistry University Hospitals Dorset NHS Foundation Trust Bournemouth UK; ^4^ Clinical Immunology University Hospital Southampton NHS Foundation Trust Southampton UK; ^5^ The Binding Site (Part of Thermo Fisher Scientific) Birmingham UK

**Keywords:** kappa light chains, multiple sclerosis, oligoclonal bands

## Abstract

**Objective:**

Kappa free light chains (KFLCs) in the cerebrospinal fluid (CSF) have a similar performance to CSF‐restricted oligoclonal bands (OCB) for multiple sclerosis (MS) diagnosis. To help with implementation, we set out to resolve several remaining uncertainties: (1) performance in a real‐world cohort and the 2024 McDonald criteria; (2) equivalence to OCB in the specific clinical scenario when demonstration of intrathecal immunoglobulin synthesis is essential for MS diagnosis; (3) which KFLC metric has the best diagnostic performance.

**Methods:**

A retrospective study of 740 cases was conducted, categorised into three groups: MS (2024 McDonald criteria), other neuroinflammatory disorders and non‐inflammatory groups. CSF and serum KFLC and albumin were assayed with immunoturbidimetry. OCB status was assessed using isoelectric focusing. Eight KFLC metrics were tested: CSF KFLC, KFLC index, three population‐based models of the upper limit for the CSF/serum kappa quotient corrected for CSF/serum albumin quotient, and their corresponding intrathecal fractions.

**Results:**

The KFLC index and the KFLC intrathecal fraction performed as well as OCB; no cases were missed when KFLC was mandatory to achieve a MS diagnosis. Intrathecal fraction computation improved the performance of the population‐based models.

**Interpretation:**

In the setting of the 2024 McDonald criteria, KFLC metrics correcting for the CSF/serum albumin quotient were equivalent to OCBs. The intrathecal fraction provided no advantage over the KFLC index, which is simpler to compute. Importantly, the KFLC index can replace OCB when CSF positivity is essential for diagnosis. We provide an explanation for KFLC's comparable diagnostic performance despite its inability to identify CSF‐only clones.

## 1 Introduction

During immunoglobulin synthesis, plasma cells produce an excess of kappa light chains which are released extracellularly as free light chains and can be quantified with highly specific assays in the serum and cerebrospinal fluid (CSF). CSF kappa free light chains (KFLCs) have a similar diagnostic performance to CSF‐restricted oligoclonal bands (OCB) in the diagnosis of multiple sclerosis (MS), confirmed in a recent meta‐analysis [1] and several studies since [2, 3, 4, 5, 6, 7, 8, 9, 10, 11, 12, 13]. Therefore the 2024 revisions of the McDonald criteria [14, 15] introduce their use as an alternative means of detecting intrathecal immunoglobulin synthesis, following a consensus recommendation [16].

While CSF‐restricted OCB have long been in use to support MS diagnosis, the 2017 revisions of the McDonald Criteria [17] allowed their presence to substitute for the requirement to fulfil dissemination in time in clinically isolated syndrome with clinical or MRI criteria for dissemination in space, and no better explanation for the clinical presentation [18]. Furthermore, CSF‐restricted OCB are a poor prognostic marker in MS [19, 20]. However, testing for CSF‐restricted OCB with isoelectric focusing (IEF) on gels followed by immunoblotting is time‐consuming and expensive, requires considerable expertise, and manual blot interpretation may be subjective [21]. Smaller laboratories may need to send their samples to larger centres where testing is usually conducted in batches to keep costs down, and the need to repeat the assay for technical reasons is not unusual. These factors collude to delay result availability, which can have knock‐on effects on time to diagnosis and treatment initiation. On the other hand, KFLC quantification is highly appealing since it can be easily conducted in most clinical laboratories and provides a rapid numerical result without the need for manual interpretation.

While the 2024 revisions of the McDonald criteria introduce the possibility of using KFLCs as an alternative to OCB as a diagnostic aid, the numerical result of the assay can be interpreted in several ways. The CSF KFLC concentration may be considered on its own, or in combination with other parameters, namely the serum KFLC concentration and blood‐CSF barrier integrity, to arrive at a more accurate estimate of intrathecal immunoglobulin synthesis. Using the CSF‐serum albumin quotient as a surrogate marker for blood‐CSF barrier integrity, several methods have been described to determine whether there is evidence of intrathecal immunoglobulin synthesis. These include a simple linear approach using a KFLC index value (calculated by dividing the CSF/serum KFLC quotient by the CSF/serum albumin quotient), upper limits of the albumin‐corrected CSF/serum KFLC quotient from three population‐derived fitted models [22, 23, 24], and the albumin‐corrected intrathecal fraction (IF) [23].

Here, we compare the performance of these different KFLC metrics in a real‐world cohort within the framework of the 2024 revisions to the McDonald criteria. The primary objectives of this study were to: (1) determine which KFLC metric has the best diagnostic performance; (2) specifically assess the substitution of OCB with KFLCs in the more relevant clinical scenario during which demonstration of intrathecal immunoglobulin synthesis is essential to make a diagnosis of MS.

## 2 Methods

### 2.1 Study Design

This retrospective study recruited inpatient and outpatient cases receiving routine clinical care at a large tertiary general hospital in the South of England, incorporating regional general neurology and specialist multiple sclerosis services, alongside other medical specialties. The study had research ethical committee (REC 11/SC/0204) and institutional (ERGO 41084.A1, SEV/0502) approvals.

Inclusion criteria were as follows: (1) a lumbar puncture and CSF/serum IEF requested as part of routine clinical care; (2) age 16 or over; and (3) access to clinical information was available. Exclusion criteria included: (1) plasma cell dyscrasia and (2) insufficient CSF/serum sample to perform all laboratory analyses.

### 2.2 Clinical Data and Diagnostic Group Assignment

The medical records of participants were reviewed to collect the following variables: age, sex, diagnosis, presence of systemic inflammatory disease, CSF protein, CSF white cell count, OCB type and immunoglobulin G (IgG) index. Cases were assigned to one of three diagnostic groups: MS, other inflammatory neurological disease (OIND) and non‐inflammatory (NI) conditions. For the purposes of this study, cases were assigned a diagnosis of multiple sclerosis based on the 2024 McDonald criteria [15], after review of clinical presentation, MRI, OCB and other investigation results at the time of diagnostic workup. Involvement of the five defined CNS topographies was enumerated after review of MRI and, where applicable, visual evoked potential or optical coherence tomography (OCT). Dissemination in time was determined based on clinical or MRI criteria. Where applicable, susceptibility weighted imaging (SWI) was reviewed for the number of central vein signs and presence of a paramagnetic rim lesion. Evidence for intrathecal immunoglobulin synthesis was based on OCB only, since one of the aims was to study the diagnostic performance of KFLC. Cases with established MS who were undergoing lumbar puncture for other indications were included in the MS group. Radiologically and clinically isolated syndromes were included in the OIND diagnostic category unless criteria for a diagnosis of MS were met according to 2024 McDonald definitions. The NI category included cases with a non‐inflammatory neurological condition and those in whom investigations did not reveal evidence of a neurological disease. All cases were under the care of a consultant neurologist. In those cases where there was residual diagnostic uncertainty, assignment to the MS, OIND or NI group was adjudicated by a professor of neurology specialising in MS and neuroinflammatory disorders.

### 2.3 Laboratory Analyses

Most CSF and serum samples were analysed for KFLCs retrospectively after thawing of frozen stored samples (*n* = 691) and the rest were analysed prospectively (*n* = 49). The retrospective cohort of samples was stored at −80°C until day of analysis. Samples were thawed at room temperature and gently mixed to ensure homogeneity prior to loading on the Optilite analyser for immunoturbidimetric quantitative free light chain, albumin and IgG measurement. The prospective cohort of samples was kept at 4°C and analysed within 7 days of collection. CSF and serum KFLC concentrations were determined using the Optilite Freelite Mx Kappa Free kit (The Binding Site, part of Thermo Fisher Scientific, LK016.M.OPT). The limit of quantification for KFLC was 0.27 mg/L; unquantifiable KFLC was equated to the absence of intrathecal immunoglobulin synthesis in all downstream analyses. CSF and serum albumin and IgG were determined using the Low Level Albumin and Low Level IgG kits (The Binding Site, part of Thermo Fisher Scientific, NK032.L.OPT and NK004.LL.OPT). The limit of quantification for albumin and IgG was 11 and 7.5 mg/L, respectively. Samples were analysed on the Optilite analyser as per manufacturer instructions and laboratory standard operating procedures, after acceptable calibration and quality control performance. All dilutions were automatically performed by the Optilite analyser as per manufacturer guidance. The coefficient of variation for all quality controls was < 10% for the duration of the analytical work.

IgG oligoclonal bands were detected by isoelectric focusing with immunofixation of both serum and CSF using the Hydragel 9 CSF isofocusing kit on the Hydrasys platform as per manufacturer guidance and standard operating procedures. Isoelectric focusing results were reported as Type 1–5 according to consensus guidelines, with Types 2 and 3 being indicative of intrathecal synthesis [25, 26].

### 2.4 Free Light Chain Metrics

Eight different KFLC metrics were considered: (1) CSF KFLC concentration (hereafter referred to as CSF KFLC), (2) KFLC index, (3) Presslauer's *Q*_KFLC_ (lim), (4) Presslauer's KFLC IF, (5) Senel's *Q*_KFLC_ (lim), (6) Senel's KFLC IF, (7) Reiber's *Q*_KFLC_ (lim), and (8) Reiber's KFLC IF.

The KFLC index was calculated with the formula: KFLC index = *Q*_KFLC_/*Q*_alb_, where *Q*_KFLC_ = CSF kappa/serum kappa and *Q*_alb_ = CSF albumin/serum albumin.

The Presslauer [22], Senel [24] and Reiber [23] models are all based on a unifying principle, namely modelling the distribution of *Q*_KFLC_ from a study population to determine an upper limit called *Q*_KFLC_ (lim) while correcting for *Q*_alb_, so that if an individual's *Q*_KFLC_ is above *Q*_KFLC_ (lim) for their *Q*_alb_, this is indicative of intrathecal synthesis. The functions used to fit the distributions in these three studies are listed below:

$$\text{Presslauer}\;2014:Q_{\text{KFLC}}\left(\lim\right)=0.9358\times {\left(Q_{\mathrm{alb}}\right)}^{0.6687}$$

$$\text{Senel}\;2019:Q_{\text{KFLC}}\left(\lim\right)=\left[14.85+\left(2.41\times \left[Q_{\mathrm{alb}}\times 10^{3}\right]\right)\right]\times 10^{-3}$$

$$\begin{array}{c} \text{Reiber}\;2019:Q_{\text{KFLC}}\left(\lim\right)\\ =\left[\left(3.27\sqrt{\left[{\left(Q_{\mathrm{alb}}\times 10^{3}\right)}^{2}+33\right]}\right)-8.2\right]\times 10^{-3} \end{array}$$

The KFLC IF is the fraction of CSF KFLC synthesised intrathecally, which so far has only been applied to Reiber's model [23]. However, in this study we derived the IF for all three models, using the same approach. KFLC IF was calculated from the respective model's *Q*_KFLC_ (lim) using the formula: KFLC IF = [KFLC_loc_/CSF kappa] × 100, where KFLC_loc_ is the amount of KFLC synthesised locally, which is in turn calculated like so: KFLC_loc_ = [*Q*_KFLC_ − *Q*_KFLC_ (lim)] × serum KFLC.

### 2.5 Statistical Analysis

Categorical data is presented as counts and percentages, continuous variables are expressed as medians and interquartile ranges. A Fisher exact test was used to evaluate differences in the categorical variables between two groups. For the continuous variables, visualisation and the Shapiro–Wilk test were used to evaluate the normality of the distributions. Differences between the two groups were tested with a Wilcoxon Rank Sum test. All analyses were 2‐tailed.

We first evaluated the diagnostic performance of KFLC for distinguishing MS from non‐MS (where non‐MS = OIND + NI). Following this, we evaluated the performance of KFLC for distinguishing MS from OIND. Cases were classified as positive or negative for each KFLC metric. Sensitivity and specificity, negative predictive value, positive predictive value, and Youden index were calculated. A receiver operating characteristic (ROC) curve was used to evaluate the diagnostic capacity (area under the curve [AUC]) of the CSF KFLC value, the KFLC index and the KFLC IF. The Youden index was used to determine the optimal threshold. The optimal threshold for the KFLC index as calculated from our data set was compared with the suggested threshold of 6.1. For comparison of Youden indices, we used non‐parametric bootstrapping with 10,000 resamples drawn with replacement, followed by calculation of 95% confidence intervals of the difference in Youden index.

Analyses were performed in R version 4.4.2 using the additional packages readxl, dplyr, writexl, stringr and pROC. Graphs were produced in GraphPad Prism version 10.4.0.

## 3 Results

740 cases were included in the analysis (MS [*n* = 148], OIND [*n* = 174], NI [*n* = 418]). Baseline characteristics are shown in Table 1. The MS group was younger than the OIND and NI groups, consisted of a higher percentage of females, and had a greater percentage of positive OCB. The median albumin quotient (*Q*_alb_) was lower in the MS group versus OIND and NI groups, but this was likely due to their younger age and larger proportion of females [27] since analysis of covariance correcting for age and male sex (which both increase *Q*_alb_), with post hoc comparisons, no longer showed a difference between MS and NI groups. The MS group had the highest median IgG index, above the reference range (0.34–0.58) reflecting intrathecal immunoglobulin synthesis [28].

**TABLE 1:** Demographic and clinical data.

Diagnosis group	MS (*n* = 148)	OIND (*n* = 174)	NI (*n* = 418)	*p* (MS vs. OIND)	*p* (MS vs. NI)
Age (year), median [IQR]	46 [34, 54]	55 [43, 68]	56 [43, 67]	< 0.001	< 0.001
Sex (female), *n* (%)	104 (70.3)	91 (52.3)	212 (50.7)	0.001	< 0.001
CSF protein (mg/L), median [IQR]	408 [335, 525.5]	515 [382, 770]	432 [336, 601]	< 0.001	0.111
CSF WBC (/mL), median [IQR]	2 [1, 4]	1 [1, 4]	1 [1, 2]	0.449	< 0.001
Albumin quotient × 10^−2^, median [IQR]	0.62 [0.47, 0.73]	0.75 [0.56, 1.29]	0.70 [0.52, 0.97]	< 0.001*	0.208*
IgG index, median [IQR]	0.77 [0.62, 1.21]	0.54 [0.49, 0.61]	0.51 [0.47, 0.56]	< 0.001	< 0.001
Positive OCB, *n* (%)	135 (91.2)	42 (24.1)	39 (9.3)	< 0.001	< 0.001
Systemic inflammatory disease, *n* (%)	6 (4.1)	15 (8.6)	39 (9.3)	0.116	0.050
Serum KFLC (mg/L), median [IQR]	14.3 [11.8, 18]	15.7 [12.5, 20.7]	16.6 [12.5, 21.3]	0.020	< 0.001

*Note:* Group comparison statistics: Wilcoxon rank sum tests for continuous data and Fisher exact tests for categorical data, except for albumin quotient, where *p* values are from post hoc comparisons after an analysis of covariance (in which diagnostic group, sex and age were explanatory variables and logarithmically transformed albumin quotient was the dependent variable)*.

Abbreviations: KFLC, kappa free light chain; MS, multiple sclerosis; NI, non‐inflammatory; OCB, oligoclonal bands; OIND, other inflammatory neurological disease; WBC, white blood cell.

### 3.1 Higher KFLC Metrics in MS

**FIGURE 1:**
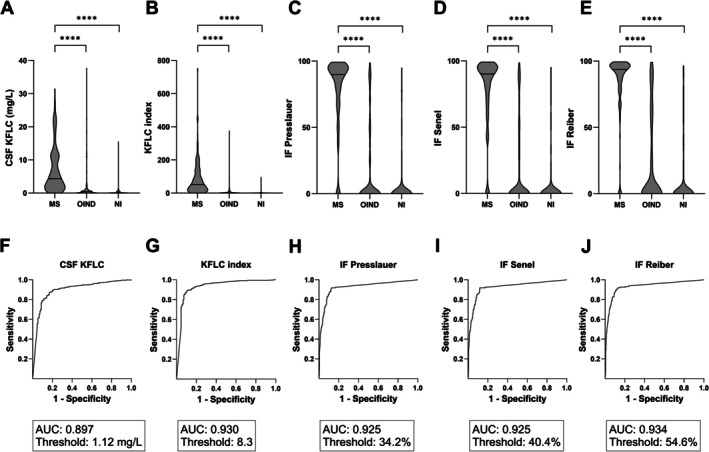
Comparison of KFLC metrics between diagnostic groups (A–E) and their receiver‐operating characteristic (ROC) curves (F–J). AUC, area under the curve; IF, intrathecal fraction (percentage); KFLC, kappa free light chain; MS, multiple sclerosis; NI, non‐inflammatory; OIND, other inflammatory neurological disease; ****p < 0.0001 (Mann–Whitney tests).

The CSF KFLC (Figure 1A), the KFLC index (Figure 1B), and the Presslauer, Senel and Reiber KFLC IF (Figure 1C–E) values were higher in the MS group (median = 4.34 mg/L, 51.8, 90%, 90.1% and 93.7%) than in the OIND group (median = 0.398 mg/L, 3.52, −39.1%, −36.3% and 8.28%, all *p* < 0.0001) or the NI group (median = 0.27 mg/L, 2.96, −66%, −58.3% and −7.35%, all *p* < 0.0001). The IFs demonstrated the best separation between MS and non‐MS groups.

### 3.2 Diagnostic Performance of KFLC Metrics

First, it was necessary to determine diagnostic threshold values for all eight KFLC metrics. The Presslauer, Senel and Reiber *Q*_KFLC_ (lim) metrics already provided this threshold by virtue of their definition as the upper limit of the *Q*_alb_‐corrected CSF/serum KFLC quotient, such that values above this limit would indicate intrathecal synthesis. Receiver‐operator curves were constructed for the other KFLC metrics (CSF KFLC, KFLC index and KFLC IFs) to determine the best threshold values discriminating between MS and non‐MS groups (Figure 1F–J).

The diagnostic performance of all KFLC metrics when discriminating between MS and non‐MS groups is shown in Table 2. The KFLC index and all three IFs had a Youden index which was similar to OCB. The CSF KFLC and Reiber's *Q*_KFLC_ (lim) metric had a lower Youden index compared to OCB. KFLC metrics which correct for the CSF/serum albumin quotient on a continuous scale (i.e., the KFLC index and all three IFs) outperformed metrics which either do not correct for the CSF/serum albumin at all (i.e., CSF KFLC) or correct for the CSF/serum albumin by dichotomising (Presslauer, Senel and Reiber *Q*_KFLC_ (lim) metrics). Since the optimal threshold for the KFLC index was calculated from our data to be 8.3 (Figure 1G) and the 2024 revisions of the McDonald criteria suggest a threshold of 6.1 [14, 15], the Youden index of the two different KFLC index thresholds was compared. The 6.1 threshold's Youden index (0.767) was lower than that of the 8.3 threshold (0.785), with 23 more false positives and three fewer false negative results out of 740 cases; the difference was not statistically significant (*p* = 0.194). There was no evidence of a sex difference in diagnostic performance of the KFLC index threshold (Table S1), in contrast to a recent report [29].

**TABLE 2:** The diagnostic performance of KFLC metrics in separating MS from non‐MS controls (*n* = 740, MS = 148, non‐MS = 592).

	Sensitivity	Specificity	Positive predictive value	Negative predictive value	Youden index		
					Value	*p* versus OCB	*p* versus CSF KFLC
KFLC index (> 8.3)	0.899	0.887	0.665	0.972	0.785	0.709	0.002
KFLC index (> 6.1)	0.919	0.848	0.602	0.977	0.767	0.731	0.009
Presslauer *Q*_KFLC_ (lim)	0.926	0.814	0.555	0.978	0.740	0.174	0.166
Senel *Q*_KFLC_ (lim)	0.926	0.809	0.548	0.978	0.735	0.128	0.242
Reiber *Q*_KFLC_ (lim)	0.939	0.755	0.489	0.98	0.694	0.001	0.766
Presslauer IF (> 34.2%)	0.919	0.872	0.642	0.977	0.791	0.529	< 0.001
Senel IF (> 40.4%)	0.919	0.882	0.660	0.978	0.801	0.309	< 0.001
Reiber IF (> 54.6%)	0.919	0.872	0.642	0.977	0.791	0.543	< 0.001
Screen‐reflex algorithm	0.899	0.894	0.679	0.972	0.792	0.285	< 0.001
CSF KFLC (> 1.12 mg/L)	0.831	0.872	0.618	0.954	0.703	0.011	NA
OCB	0.912	0.863	0.625	0.975	0.775	NA	0.011

*Note:* In the screen‐reflex algorithm simulation, KFLC index values < 3.5 or > 20 were considered to be the final result and the OCB result was utilised for KFLC index values in the 3.5–20 grey zone, such that isoelectric focusing was needed in only 18% of cases.

Abbreviations: IF, intrathecal fraction; KFLC, kappa free light chain; lim, upper limit of *Q*_KFLC_ derived from model correcting for the CSF/serum albumin quotient; NA, not applicable; OCB, oligoclonal bands; *Q*_KFLC_, CSF/serum KFLC.

A more clinically relevant scenario is differentiating MS from other neuro‐inflammatory disorders, so the diagnostic performance for this comparison is shown in Table 3. As expected, all metrics performed better in the MS versus non‐MS comparison (Table 2) than the MS versus OIND comparison (Table 3). In this clinical setting, the Youden indices for all KFLC metrics were similar to OCB, except Reiber's *Q*_KFLC_ (lim) metric, which performed less well. Once again, the KFLC index and all three IFs outperformed the CSF KFLC and the Presslauer, Senel and Reiber *Q*_KFLC_ (lim) metrics.

**TABLE 3:** The diagnostic performance of KFLC metrics in separating MS from OIND (*n* = 322, MS = 148, OIND = 174).

	Sensitivity	Specificity	Positive predictive value	Negative predictive value	Youden index		
					Value	*p* versus OCB	*p* versus CSF KFLC
KFLC index (> 8.3)	0.899	0.805	0.796	0.903	0.703	0.382	0.020
KFLC index (> 6.1)	0.919	0.741	0.751	0.915	0.660	0.761	0.309
Presslauer *Q*_KFLC_ (lim)	0.926	0.695	0.721	0.917	0.621	0.195	0.947
Senel *Q*_KFLC_ (lim)	0.926	0.695	0.721	0.917	0.621	0.218	0.943
Reiber *Q*_KFLC_ (lim)	0.939	0.632	0.685	0.924	0.571	0.013	0.206
Presslauer IF (> 34.2%)	0.919	0.776	0.777	0.918	0.695	0.498	0.029
Senel IF (> 40.4%)	0.919	0.799	0.795	0.921	0.718	0.184	0.003
Reiber IF (> 54.6%)	0.919	0.776	0.777	0.918	0.695	0.504	0.028
Screen‐reflex algorithm	0.899	0.793	0.787	0.902	0.692	0.363	0.050
CSF KFLC (> 1.12 mg/L)	0.831	0.793	0.774	0.847	0.624	0.260	NA
OCB	0.912	0.759	0.763	0.91	0.671	NA	0.260

*Note:* In the screen‐reflex algorithm simulation, KFLC index values < 3.5 or > 20 were considered to be the final result and the OCB result was utilised for KFLC index values in the 3.5–20 grey zone.

Abbreviations: IF, intrathecal fraction; KFLC, kappa free light chain; lim, upper limit of *Q*_KFLC_ derived from model correcting for the CSF/serum albumin quotient; NA, not applicable; OCB, oligoclonal bands; *Q*_KFLC_, CSF/serum KFLC.

Arguably, the patients for whose care the outcome of CSF/serum KFLC evaluation is most critical are those in whom evidence of intrathecal immunoglobulin production is essential to fulfil the criteria for MS diagnosis and thereby access treatment. Therefore, we conducted an analysis of these cases in our study (*n* = 29, Table 4). In all 29 cases, who were diagnosed on the basis of CSF‐restricted OCB, all kappa metrics were positive with the exception of CSF KFLC, which missed 3/29 cases.

**TABLE 4:** The diagnostic performance of KFLC metrics in separating MS cases where positive OCB were essential to fulfil the 2024 McDonald criteria for MS diagnosis, from OIND (*n* = 203, MS = 29, OIND = 174).

	Sensitivity	Specificity	Positive predictive value	Negative predictive value	Youden index
KFLC index (> 8.3)	1	0.805	0.460	1	0.805
KFLC index (> 6.1)	1	0.741	0.392	1	0.741
Presslauer *Q*_KFLC_ (lim)	1	0.695	0.354	1	0.695
Senel *Q*_KFLC_ (lim)	1	0.695	0.354	1	0.695
Reiber *Q*_KFLC_ (lim)	1	0.632	0.312	1	0.632
Presslauer IF (> 34.2%)	1	0.776	0.426	1	0.776
Senel IF (> 40.4%)	1	0.799	0.453	1	0.799
Reiber IF (> 54.6%)	1	0.776	0.426	1	0.776
Screen‐reflex algorithm	1	0.793	0.446	1	0.793
CSF KFLC (> 1.12 mg/L)	0.897	0.793	0.419	0.979	0.69
OCB	1	0.759	0.408	1	0.759

*Note:* In the screen‐reflex algorithm simulation, KFLC index values < 3.5 or > 20 were considered to be the final result and the OCB result was utilised for KFLC index values in the 3.5–20 grey zone.

Abbreviations: IF, intrathecal fraction; KFLC, kappa free light chain; lim, upper limit of *Q*_KFLC_ derived from model correcting for the CSF/serum albumin quotient; OCB, oligoclonal bands; *Q*_KFLC_, CSF/serum KFLC.

### 3.3 Screen‐Reflex Algorithm

**FIGURE 2:**
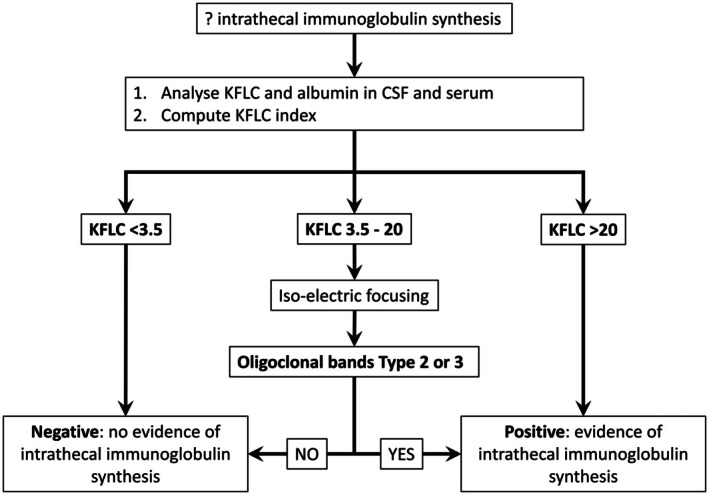
Screen‐reflex algorithm.

Next, we reasoned that some centres may not be keen to switch completely from OCB to KFLC, and may prefer a screening approach with KFLC followed by IEF in a defined subset of cases, especially given investigators suggesting this approach [2, 3, 9, 10, 11, 12, 13]. Hegen et al. [30] have recently suggested a grey zone between KFLC index values of 3.5 and 20 for reflex testing with IEF, since values outside this range showed a predictive value exceeding 95% to detect OCB negativity and positivity. In our data, KFLC index values of < 3.5 and > 20 had a 95% negative predictive value and a 96% positive predictive value for OCB, respectively. Hence, an in silico simulation of a screen‐reflex algorithm was conducted (Figure 2). If the KFLC index was lower than 3.5 or higher than 20, this was considered to be the final result. For KFLC index values between 3.5 and 20, the OCB result was utilised. This screen‐reflex algorithm reduced OCB testing from 100% to 18% of all cases during the period of study. Its Youden index was not different from that of OCB (*p* = 0.285), the KFLC index (*p* = 0.750), and all three IFs (*p* = 0.912, 0.646 and 0.900). Four out of the seven KFLC‐negative OCB‐positive MS cases were in the grey zone, but in none of these was CSF positivity required to achieve a MS diagnosis according to the 2024 revisions of the McDonald criteria.

### 3.4 Cases Discordant for KFLC Metric and OCB

**FIGURE 3:**
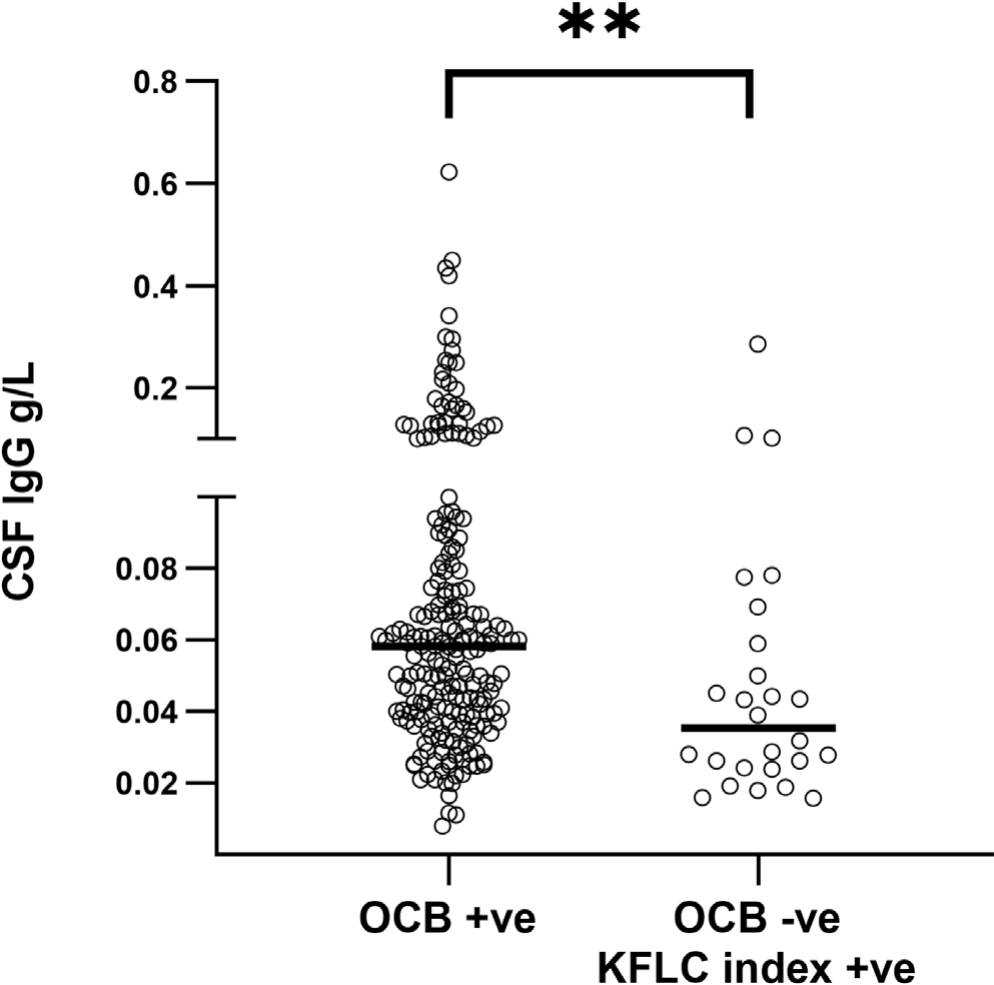
Cerebrospinal fluid immunoglobulin G concentration in OCB‐negative KFLC index‐positive cases versus OCB‐positive cases. −ve, negative; +ve, positive; CSF, cerebrospinal fluid; IgG, immunoglobulin G; KFLC, kappa free light chain; OCB, oligoclonal bands. Horizontal line represents median. **p < 0.01 (Mann–Whitney test).

Cases which were discordant for KFLC (according to the index and IFs) and OCB were studied in more detail (Table 5). One possible reason for the OCB‐negative KFLC‐positive discordance is a low level of CSF immunoglobulin G which makes visual detection of OCB bands challenging due to a low signal‐to‐noise ratio. Indeed, we found that CSF immunoglobulin G was significantly lower in OCB‐negative KFLC‐positive cases when compared to OCB‐positive cases (Figure 3, *p* < 0.01). Although this could imply that KFLC is more sensitive than OCB for MS diagnosis, this was not the case (Tables 2 and 3). A closer examination of Table 5, focusing on cases with MS, revealed that the discordant MS cases were as likely to be OCB‐negative KFLC‐positive as vice versa. Hence the extra OCB‐negative KFLC‐positive MS cases diagnosed were offset by the OCB‐positive KFLC‐negative cases, explaining why the overall sensitivity of KFLC and OCB is similar.

**TABLE 5:** Cases discordant for KFLC metrics and OCB, with their diagnosis.

	OCB+ and KFLC metric–	OCB– and KFLC metric+	*p*
OCB and KFLC index			
Total	43	27	1.0
MS	7	5	
Not MS	36	22	
OCB and Presslauer IF			
Total	36	32	0.725
MS	4	5	
Not MS	32	27	
OCB and Senel IF			
Total	40	30	0.483
MS	4	5	
Not MS	36	25	
OCB and Reiber IF			
Total	38	34	0.727
MS	4	5	
Not MS	34	29	

*Note:* *p* value is from a Fisher exact test.

Abbreviations: IF, intrathecal fraction; KFLC, kappa free light chain; OCB, oligoclonal bands.

## 4 Discussion

Clarity regarding the comparative real‐world performance of KFLC metrics is needed to enable centres worldwide to implement KFLC, now integrated into the 2024 revisions of the McDonald criteria [15], as a more practical, rapid and efficient alternative to OCB. The equivalent diagnostic performance of KFLC and OCB has been known for more than a decade, but the multiplicity of CSF KFLC metrics, as well as the complexity of generating them, may have impeded the uptake of KFLC as an aid to MS diagnosis. This study provides reassurance that all KFLC metrics, other than CSF KFLC, can replace OCB in the specific clinical situation when OCB is essential to make an MS diagnosis. Our data, alongside the extensive body of research employing the KFLC index, shows a lack of a discernible advantage conferred by alternative KFLC metrics, and given its simplicity, the KFLC index should be established as the preferred metric, as advised in a companion paper [14] to the 2024 revisions of the McDonald criteria [15].

In all clinical scenarios, intrathecal fraction computation improved the performance of the population‐based models. This can be explained by the fact that, while *Q*_KFLC_ (lim) metrics correct for blood‐CSF barrier integrity, they simply define an upper limit of a reference range without taking advantage of the fact that intrathecal immunoglobulin synthesis is much higher in MS than in OIND or NI groups (Figure 1A–E).

The best performing KFLC metrics are those correcting for blood‐CSF barrier integrity while taking into account the level of intrathecal immunoglobulin synthesis: the KFLC index and the three IFs (based on Presslauer, Senel and Reiber models). The KFLC index is endowed with a simplicity which is intuitive and easy to grasp, while its calculation is straightforward to embed in laboratory information operating systems for immediate clinical reporting.

The uncorrected CSF KFLC performed less well than OCB. In a study which used a cut‐off (1.0 mg/L) very similar to the cut‐off used here (1.12 mg/L), CSF KFLC approximated the performance of OCB when distinguishing MS from non‐MS groups [31]. Here, we focussed on the clinical scenario where intrathecal immunoglobulin synthesis is essential for MS diagnosis and found that CSF KFLC missed 3/29 (10%), while all other KFLC metrics did not miss any MS diagnoses. Additionally, the Youden index of CSF KFLC was significantly lower than that of the KFLC index and the three IFs (Tables 2 and 3). CSF KFLC has the advantage of being a single measurement, while other CSF metrics rely on four measurements (two in CSF and two in serum) which may create challenges associated with cost, consistency and reproducibility. A potential disadvantage of the CSF KFLC is that the diagnostic threshold value may vary depending on the analytical method used, while quotients such as the KFLC index may have the potential to mathematically eliminate systemic biases between different assays. Finally, as reviewed recently by Deisenhammer and colleagues [14], the diagnostic performance of CSF KFLC decreases when present at low concentrations, since intrathecally‐produced KFLC are eclipsed by fluctuations in blood‐derived CSF KFLC.

OCB provide two items of information about intrathecal immunoglobulin synthesis which KFLC do not: (1) clonality, that is, whether monoclonal, oligoclonal or polyclonal (2) the differential presence of clones in CSF and serum (i.e., CSF alone, serum alone, in both CSF and serum). Both are characteristics in keeping with our understanding of MS pathology, including features such as terminal B cell differentiation within the CNS compartment and epitope spreading with time. While several KFLC metrics can quantify intrathecal synthesis, none can locate clones in CSF and serum; this is important since in the presence of systemic inflammation with an immunoglobulin response in both compartments, it may be difficult to detect CSF‐only immunoglobulin production using a quantitative assay, while a CSF‐only clone is clearly visible using IEF. However, these theoretical concerns do not play out to be a significant factor in real‐world clinical practice, and once again this study has confirmed previous findings that CSF KFLC has comparable diagnostic performance to OCB, especially when corrected by the *Q*_alb_. Here we provide an explanation for this phenomenon, which so far has been unexplained—the OCB‐positive KFLC‐negative MS cases are offset by OCB‐negative KFLC‐positive MS cases in whom CSF immunoglobulin G levels are low (Table 5, Figure 3), such that overall diagnostic performance of OCB and KFLC is comparable.

Nevertheless, there may still be resistance to adopt KFLC as an alternative or substitute for OCB, because of the theoretical concerns discussed above and the long‐established nature of OCB in MS diagnosis. In addition there are two reasons driving the need to ensure the continued availability of IEF. First, completely replacing OCB with KFLC in neuroimmunology laboratories will result in deskilling, and IEF is useful in other clinical scenarios which are less frequent than multiple sclerosis, such as other neuroinflammatory conditions and certain neuro‐infections. Second, although all KFLC‐negative OCB‐positive MS cases in our dataset did not require CSF positivity to achieve a diagnosis of MS within the framework of the 2024 McDonald criteria, there may be cases where the clinician may still request CSF examination in these cases, for example if there are soft red flags. For these reasons, we have simulated a screen‐reflex algorithm using the KFLC index for screening followed by reflex testing for OCB if the KFLC index falls within the grey zone [30] (Figure 2). This approach has the potential to dramatically decrease the number of samples needing IEF (by 82%), thereby still making significant time and cost savings. We hope that the data presented here, and the above explanation providing a rational mechanism underlying the non‐inferiority of KFLC versus OCB, despite the fact that KFLC is unable to ascertain clonality, will help increase acceptance of KFLC.

The 2024 revisions to the McDonald criteria define positive CSF as presence of CSF‐restricted OCBs (Types 2 or 3) and/or KFLC index above 6.1 [14, 15]. This is based on a meta‐analysis of 32 studies, which determined an optimum threshold for the KFLC index of 6.1, with a range of 2.4 to 20 [1]. The studies included in this meta‐analysis were very varied, employing different laboratory kits, populations and comparison groups, hence the wide range. While the KFLC index threshold value of 8.3 had a higher Youden index compared to the 6.1 threshold in our study, the difference was not statistically significant. A similar observation was made by Hegen et al., who found that minor variations in site‐specific KFLC index cut‐offs between 4.6 to 7.5 did not affect diagnostic performance [7]. This is reassuring since it means that minor variations in the KFLC index threshold do not have a large clinical impact. However, laboratories employing the same KFLC assay as that used here may decide to take advantage of our study and adopt the 8.3 KFLC index threshold, given its higher Youden index.

This study has a number of strengths and innovations, including a thorough side‐by‐side comparison of all KFLC metrics, the application of Reiber's IF methodology to Presslauer and Senel models, the large sample size, the real‐world setting, the evaluation within the framework of the 2024 revisions to the McDonald diagnostic criteria, the consideration of clinically relevant scenarios including when evidence of intrathecal immunoglobulin synthesis was pivotal in making a MS diagnosis, and the provision of a rational explanation for the excellent performance of KFLC despite being clonally agnostic. However, some limitations are worth noting. We only used one type of KFLC assay, and the study was conducted in one country. While this may limit the generalisability of results, one is reassured by the fact that the optimum KFLC index threshold value of 8.3 is very close to that of the meta‐analysis referred to above [1]. Another limitation was that this study was retrospective, and while all clinically isolated syndrome, OIND and MS cases were reviewed to determine whether they fulfilled the 2024 revisions of the McDonald diagnostic criteria, eight cases in whom a diagnosis of MS could have been made in the presence of central vein signs or a paramagnetic rim lesion did not have susceptibility weighted imaging. While using samples that were frozen and thawed for KFLC assay may be considered a limitation, this is mitigated by experimental studies showing that KFLC determination is not affected by a single freeze–thaw cycle [32, 33]. One has to be mindful that OCB positivity was utilised in some cases to arrive at a diagnosis of MS, so their diagnostic performance indices in Tables 2, 3, 4 are likely to be somewhat inflated; however, this should strengthen our reassurance that KFLC can indeed substitute OCB.

In conclusion, this real‐world study supports previous findings that KFLC is a reliable alternative to OCB as a test for intrathecal immunoglobulin synthesis and extends this finding to the setting of the 2024 revisions to the McDonald diagnostic criteria. All *Q*_alb_ corrected KFLC metrics can be used safely when a positive result is essential for making an MS diagnosis. The KFLC index and KFLC IFs performed equally well, and since the KFLC index is very simple to calculate and more intuitive, it should be the KFLC metric of choice.

## Author Contributions

I.G., D.G., V.I., and A.M.E.W. contributed to the conception and design of the study. M.M.L., R.C., S.K., M.B., K.I.C., W.G., M.E., D.T.S., A.M.E.W., and I.G. contributed to the acquisition and analysis of data. M.M.L., K.I.C., and I.G. drafted a significant portion of the manuscript and/or figures.

## Funding

This work was supported by the Binding Site (Part of Thermo Fisher Scientific) and National Institute for Health and Care Research.

## Conflicts of Interest

I.G. received the following funding in the last 36 months: research grants from Medical Research Council UK, National Institute for Health and Care Research, MS Society UK, Wessex Medical Research, Independent Research Fund Denmark, Rosetrees Trust, Guarantors of Brain, Kedrion, The Binding Site (Part of Thermo Fisher Scientific), and Evgen; speaker honoraria and travel funding from Novartis. D.G. and V.I. are employed by Binding Site (Part of Thermo Fisher Scientific). The other authors declare no conflicts of interest.

## Supporting information


**Table S1:** The diagnostic performance of KFLC index metrics across gender (*n* = 740, MS = 148, non‐MS = 592). Abbreviations: KFLC = kappa free light chain.

## Data Availability

The data that support the findings of this study are available from the corresponding author upon reasonable request, subject to institutional and ethical approvals.
