# Supplementary material for: Real‐World Performance of CSF Kappa Free Light Chains in the 2024 McDonald Criteria
Source: Ann Clin Transl Neurol. 2026 Jan 7;13(6):1186–95. doi: 10.1002/acn3.70300 (PMC13251450; doi:10.1002/acn3.70300)
Supplement: Supplementary file 1 — Table S1: The diagnostic performance of KFLC index metrics across gender (n = 740, MS = 148, non‐MS = 592). Abbreviations: KFLC = kappa free light chain. [file ACN3-13-1186-s001.docx]

**Supporting Information for:**

**Real-world performance of CSF kappa free light chains in the 2024 McDonald criteria**

Maya M Leibowitz BA MB BChir MRCP^1,2,#^, Ryan Cooper BSc MSc FRCPath^3,#^, Stefania Kaninia MD MSc MRCP^1,2^, Kathryn I Challis BSc (Hons) MRes MSc^4^, Will Greenway BSc MRes MSc^4^, Maria Bonello MD MRCP FEBN^1,2^, Mahmoud Elbahnasawi BA MB BChir^1,2^, Deborah T Shode MD^1,2^, Dominika Gajdasik PhD^5^, Victor Iliev PhD^5^, Alison M E Whitelegg BSc MSc PhD FRCPath^4,*^, Ian Galea MD FRCP PhD^1,2,*^

^1^ Clinical Neurosciences, Clinical & Experimental Sciences, Faculty of Medicine, University of Southampton, Southampton, UK

^2^ Wessex Neurological Centre, University Hospital Southampton NHS Foundation Trust, Southampton, UK

^3^ Clinical Biochemistry, University Hospitals Dorset NHS Foundation Trust, Bournemouth, UK

^4^ Clinical Immunology, University Hospital Southampton NHS Foundation Trust, Southampton, UK

^5^ The Binding Site (part of Thermo Fisher Scientific), Birmingham, UK

Supplementary Table S1

**Table S1.** **The diagnostic performance of KFLC index metrics across gender** (n = 740, MS = 148, non-MS = 592). Abbreviations: KFLC = kappa free light chain

|  | **KFLC index > 8.3** | | | **KFLC index > 6.1** | | |
| --- | --- | --- | --- | --- | --- | --- |
|  | **Male** | **Female** | **p** | **Male** | **Female** | **p** |
| Sensitivity | 0.864 | 0.913 | 0.380 | 0.886 | 0.933 | 0.342 |
| Specificity | 0.907 | 0.868 | 0.154 | 0.872 | 0.825 | 0.136 |
| Positive predictive value | 0.585 | 0.704 | 0.111 | 0.513 | 0.647 | 0.062 |
| Negative predictive value | 0.978 | 0.967 | 0.602 | 0.981 | 0.973 | 0.772 |
| Youden index | 0.770 | 0.781 | 0.875 | 0.758 | 0.758 | 0.97 |
